# Evaluation of the fusion inhibitor P3 peptide as a potential microbicide to prevent HIV transmission in women

**DOI:** 10.1371/journal.pone.0195744

**Published:** 2018-04-18

**Authors:** Inês Bártolo, Ana Rita Diniz, Pedro Borrego, João Pedro Ferreira, Maria Rosário Bronze, Helena Barroso, Rui Pinto, Carlos Cardoso, João F. Pinto, Rafael Ceña Diaz, Pilar Garcia Broncano, Maria Angel Muñoz-Fernández, Nuno Taveira

**Affiliations:** 1 Research Institute for Medicines (iMed.ULisboa), Faculdade de Farmácia da Universidade de Lisboa, Lisboa, Portugal; 2 Centro de Administração e Políticas Públicas (CAPP), Instituto Superior de Ciências Sociais e Políticas (ISCSP) da Universidade de Lisboa, Lisboa, Portugal; 3 Centro de Investigação Interdisciplinar Egas Moniz (CiiEM), Instituto Superior de Ciências da Saúde Egas Moniz, Caparica, Portugal; 4 Laboratório de Análises Clínicas Dr. Joaquim Chaves, Joaquim Chaves Saúde, Miraflores-Algés, Portugal; 5 Laboratorio InmunoBiología Molecular, Hospital General Universitario Gregorio Marañón, Madrid, Spain; 6 Instituto de Investigación Sanitaria Gregorio Marañón. Spanish HIV-HGM Biobank, Madrid, Spain; 7 Networking Research Center on Bioengineering, Biomaterials and Nanomedicine (CIBER-BBN), Madrid, Spain; Scripps Research Institute, UNITED STATES

## Abstract

Microbicides are an important strategy for preventing the sexual transmission of HIV but, so far, the most advanced tenofovir-based microbicides have had modest efficacy. This has been related to adherence problems and high prevalence of tenofovir-resistant HIV-1 strains. P3 is a new peptide with potent activity against HIV that may be a good microbicide candidate. In this work P3 was formulated in a gel of hydroxyethyl cellulose and its activity, stability and safety profile in Balb/c mice were evaluated. HIV infection was fully blocked by a 1.5% gel containing P3 at the IC_90_ (366.4 nM) concentration. The antiviral activity did not change at 4°C during 4 months and at 25, 37 and 65°C for 1 week. P3 was stable and fully functional at acidic pH up to 24h, under different concentrations of hydrogen peroxide and in the presence of genital fluids up to 48h. P3 had no antibacterial activity and did not affect sperm motility and vitality. Finally, P3 didn’t cause significant alterations in the vaginal epithelium of Balb/c mice at 0.06 (456.8 μM) and 0.2 mg/day (1522.7 μM) doses. These findings indicate that P3 is an excellent candidate for further development as a microbicide gel for the prevention of HIV transmission in women.

## Introduction

At the end of 2015 almost 37 million people were living with HIV [1, 2]. Sub-Saharan Africa encompasses the majority of the infected population (25.5 million). Women, who acquire the virus mainly by heterosexual exposure, now account for approximately half of the infected population worldwide (17.8 million) and greater than 58% in sub-Saharan Africa.

New HIV infections have fallen by 38% since 2001 [1]. Nonetheless, an estimated 2.1 million people became newly infected with HIV in 2015. The control of HIV pandemic requires the development and use of safe and effective prevention methods. Condoms can be an effective barrier against HIV transmission but its use is unreliable and often not within the woman’s control [3]. Oral pre-exposure prophylaxis (PrEP) with TDF or TDF/FTC can play an important role in HIV prevention in women [4, 5]. Its effectiveness is highly dependent on adherence levels and, because of the TDF component, it can cause a decrease in spine and total hip bone mineral density [6] and a decline in renal function [7, 8]. Moreover, the time from initiation of daily tenofovir disoproxil/emtricitabine (TDF/FTC) to maximal protection against HIV infection is still unknown. Topical PrEP using microbicides is an additional strategy for preventing the transmission of HIV through sexual intercourse. Microbicides are products (rings, gels, films or inserts) used topically, either vaginally or rectally, to prevent entry and/or replication of HIV in the cells of those mucosae [9, 10]. The ideal microbicide candidate for HIV prevention should observe the following requirements: be active on HIV-1 and HIV-2, easy to use and discrete, painless to the user, cost-effective, and safe and well tolerated for chronic administration [9, 10]. It should allow self-administration with minimal interference in body function and daily life. Finally, it should provide long-standing protection and maintain activity in the presence of vaginal fluid and semen. Most of the first generation microbicides, which include surfactants, polyanionic and acidifying agents, were abandoned due to safety issues and lack of efficacy [11–15]. The new generation of microbicides contain antiretroviral drugs (ARV) with potent activity against HIV and that, in most cases, are being used to treat HIV infection [12]. The CAPRISA 004 trial carried out on women from South Africa using a daily dose of 1% TDF vaginal gel reported a reduction of 39% of HIV acquisition; impressively, in high adherents (>80%) HIV acquisition was reduced by 54% [1]. However, in the VOICE study, a confirmatory study carried out in women from South Africa, Uganda and Zimbabwe the rate of HIV-1 acquisition was only reduced by 15%; this was associated with very low adherence [16]. Likewise, in the FACTS 001 trial which took place in South Africa the effectiveness of the TDF gel (1%) was null when the entire population of trial participants was analyzed [17]. More recently in the ASPIRE study the risk of acquiring HIV was reduced up to 37% using a dapivirine-infused vaginal ring [18]. This phase 3 clinical trial was conducted in women from Malawi, South Africa, Uganda and Zimbabwe. Interestingly, while no protection was observed in women younger than 21 years which appeared to use the ring inconsistently, in women older than 21 years the dapivirine ring reduced the risk of HIV infection by 56%. Taken together, these results indicate that the current microbicides confer partial protection from HIV in women in sub-Saharan Africa.

As recommended by the 2013 WHO guidelines, TDF is increasingly being used in first-line regimens with efavirenz and lamivudine or emtricitabine for the treatment of adult HIV-1 infection [19]. TDF treatment selects for TDF-resistant HIV-1 strains which already reach high proportions in low- and middle-income countries [20]. A TDF-based microbicide will likely be ineffective against such TDF-resistant strains. Moreover, its regular use may promote the selection and dissemination of TDF- resistant strains that will not respond to TDF-based therapeutic regimens [11]. Hence, one priority in this field is to find new microbicide candidates that interfere with the life cycle of HIV-1 and HIV-2 but are not based on antiretroviral drugs in current use. Recent studies indicate that entry inhibitors may be good microbicides as they effectively prevent vaginal HIV-1 transmission in humanized mice models [21].

We have recently described a new fusion inhibitor peptide named P3 based on ancestral sequences of the transmembrane glycoproteins of HIV-2 and SIV [22]. P3 forms a typical α-helix structure in solution, binds strongly to its target in the transmembrane glycoprotein and potently inhibits both HIV-1 and HIV-2 replication [22]. The high α-helical and reduced random coil contents of P3 are thought to determine its high target binding affinity, and predict that P3 should be more resistant to degradation by proteolytic cleavage in biologic fluids than other fusion inhibitor peptides [23]. Combined, these features make P3 an attractive microbicide candidate for HIV prevention. In the current study we evaluated the stability and antiviral function of P3 under different conditions of pH, temperature and oxidative stress, as well as its antiviral activity in a gel of hydroxyethyl cellulose (HEC). We also evaluated the safety profile of P3 in Balb/C mice.

## Material and methods

### Therapeutically active compound

P3 is a peptide with 34 amino acid residues and a molecular weight of 4,379.87 g/mol whose sequence overlaps the N-terminal pocket-binding region and core of the heptad repeat (HR) 2 region in the HIV/SIV transmembrane glycoprotein (P3 amino acid sequence: WQEWEQQVRYLEANISQRLEQAQIQQEKNMYELQ). P3 was derived from ancestral sequences of the transmembrane glycoproteins of HIV-2 and SIV and potently inhibits HIV-1 and HIV-2 replication (mean IC_50_ for HIV-1, 11.0 nM; mean IC_50_ for HIV-2, 63.8 nM) [22]. The P3 peptide was produced commercially by Genemed Synthesis (San Antonio, Texas, USA). It was modified with the N-terminus acetylated and the C-terminus as a carboxamide, the salt form being acetate. Reverse-phase high-pressure liquid chromatography (HPLC) was used for purification (>95%) and mass spectrometry for confirmation analysis.

### Cell lines and virus

TZM-bl cells (AIDS Research and Reference Reagent Program, National Institutes of Health, USA) were cultured in complete growth medium that consists of Dulbecco’s minimal essential medium (DMEM) supplemented with 10% fetal bovine serum (FBS), 100 U/ml of penicillin-streptomycin (Gibco/Invitrogen, USA), 1mM of sodium pyruvate (Gibco/Invitrogen, USA), 2mM of L-glutamine (Gibco/Invitrogen, USA) and 1mM of non-essential amino acids (Gibco/Invitrogen, USA).

Peripheral blood mononuclear cells (PBMCs) from healthy individuals (blood donors) were separated by Ficoll-Paque PLUS (GE Healthcare, Waukesha, WI, USA) density gradient centrifugation and stimulated for 3 days with 5 μg/ml of phytohemaglutinin (PHA; Sigma-Aldrich, St. Louis, MO, USA). PBMCs cultures were maintained in RPMI-1640 medium supplemented with 10% FBS, 100 U/ml of penicillin-strepotmycin, 2mM of L-glutamine (Gibco/Invitrogen, USA) 0.3 mg/ml of gentamicin (Gibco/Invitrogen, Carlsbad, CA, USA), 5μg/ml of polybrene (Sigma-Aldrich, St. Louis, MO, USA) and 20U/ml units of recombinant interleukin-2 (Roche, Basel, Switzerland). All cell cultures were maintained at 37°C in 5% CO_2_.

The clinical isolate 93AOHDC50 used in this study was previously isolated, titrated and characterized for co-receptor usage [24]. The 50% tissue culture infectious dose (TCID_50_) of the virus was determined in a single-round viral infectivity assay using a luciferase reporter gene assay in TZM-bl cells [24, 25] and calculated using the statistical method of Reed and Muench [26].

### Seminal plasma and vaginal fluid simulant

Semen samples were collected in a sterile container from seven HIV-1-seronegative healthy individuals by masturbation. The volunteers had no recent history of sexual transmitted infections, no urogenital abnormalities, and abstinence from sexual intercourse for 48h prior to collection. The semen samples were allowed to liquefy for 30–45 min at room temperature and seminal plasma (SP) was obtained by centrifuging pooled whole semen at 1200 g for 10 min at 4°C. The SP was filtered and stored in aliquots at -80°C until use.

Vaginal fluid simulant (VFS) was prepared as described elsewhere [27]. 1 L of VFS contained the following reagents: 3.51g of NaCl; 1.400g of KOH; 0.222g of Ca(OH)2; 0.018g of bovine serum albumin; 2.0g of lactic acid; 1.00g of acetic acid; 0.16g of glycerol; 0.4g of urea and 5.0g of glucose. The pH was adjusted to 4.2 using HCl (1M).

SP and VFS cytotoxicity was assessed in TZM-bl cells. Cells were cultured in serial-fold dilutions of the biologic fluids and cellular viability was investigated using the alamarBlue assay [28].

### Stability and antiviral activity of P3 in the presence of biologic fluids

The antiviral activity of P3 in the presence of SP and VFS was determined with a single-round viral infectivity assay using TZM-bl reporter cells, as previously described [24]. Briefly, TZM-bl cells were plated (1x10^4^ cells/well) in a 96-plate and incubated overnight at 37°C with 5% CO_2_. In the following day, the IC_90_ concentration of the peptide (366.4 nM) was added to each well in a total volume of 100 μl of complete growth medium in the presence of either 0.5% or 1% (v/v) SP. The same procedure was done with VFS and with SP+VFS. Cells were then infected with 200 TCID50 (in 100 μl) of HIV-1 isolate 93AOHDC250 and incubated for 48h before measuring the luciferase activity with the Pierce Firefly Luc One-Step Glow Assay Kit (ThermoFisher Scientific, Rockford, USA) according to the manufacturer’s instructions. At least two independent experiments, each in triplicate, were performed for each antiviral activity analysis. For these experiments, the positive control was made of cells plus virus, the negative control was made of cells plus complete growth medium, compound controls were made of cells plus P3 alone or P3 in SP, VFS or SP+VPS, and the biological fluids control were made of cells plus SP, VFS or SP+VFS.

The long-term stability of P3 was evaluated in the presence of SP [0.5% or 1% (v/v)] or VFS at 37°C for a period of 24h, 48h and 1 week before performing the assay, as described above. In all cases, P3 was considered stable in the presence of biologic fluids if its antiviral activity was unaffected by SP, VFS and SP+VFS. In addition, peptide concentration was determined after exposure to the above conditions by mass spectrometry as described below.

### Liquid chromatography and mass spectrometry (LC-MS/MS) analysis

Individual standard solutions (0.25–250 ppm) of P3 were prepared in acetonitrile (99.9% LC–MS) (J.T.Baker, Deventer, the Netherlands) in the presence of SP (0.5 or 1.0%), VFS or growth medium (GM) and analyzed after 24h, 48h and 1 week. In order to precipitate the proteins in the biological fluids, 1 ml of acetonitrile (1:1) was added to 1 ml of sample and samples were centrifuged at 13,000 rpm for 10 min and filtered (0.20 mm PVDF membrane; Chromafil®Xtra from Macherey-Nagel®) just before analysis, All samples were analyzed as triplicates. Experiments were performed to confirm that this procedure did not interfere with the concentration of the analyte in the standard solutions.

#### Liquid chromatography (LC)

The LC system was a Waters® Alliance 2695 (Waters®) equipped with a quaternary pump, solvent degasser, auto sampler and column oven, coupled to a Photodiode Array Detector Waters 996 PDA (Waters®) scanning from 210 to 600 nm. The separation was performed on a reversed phase column LiChrospher® 100 RP-18 (10 x 4.0mm; Merck®, Germany) operating at 35 ^o^C, using an injection volume of 10 μl. The mobile phase consisted of water [Ultra-pure water (18.2MΩ.cm) from a Millipore-Direct Q3 UV system (Millipore, USA)] with TFA 0.1% (v/v) (98% p.a.; Merck®, Germany) (A) and acetonitrile with TFA 0.1% (v/v) (B) at a flow rate of 0.30 ml/min. The following gradient was used: 70% of A as initial conditions, 70% A to 30% A for 7 min. A cleaning step was performed using 100% of acetonitrile for 1 min and finally the column was re-equilibrated with 70% of eluent A for 5 min.

### Tandem mass spectrometry

A triple quadrupole mass spectrometer MicroMass® Quattro micro (Micromass®, Waters®) with an electrospray ionization source (ESI) was coupled to the LC system, described above. The ion source conditions were optimized using a standard solution directly injected in the ion source: temperature 120°C, capillary voltage 3.0 kV and cone voltage 50 V. The compound was ionized in positive ion mode and the spectra, in Full Scan, were recorded in the range m/z 100–1700. All analyses were performed in multiple reactions monitoring (MRM) mode in order to achieve a high selectivity and sensitivity. For the MS/MS experiments, collision energy was optimized (55 eV) to maximize [precursor ion > product ion] the transitions signal. Three transitions were considered: [1460>229]; [1460>258]; [1460>486]. The optimized conditions were confirmed when performing the LC–MS/MS analysis of the standard solutions.

High purity nitrogen (N_2_) was used both as drying and nebulizing gas. Ultrahigh-purity Argon (Ar) was used as collision gas. MassLynx^TM^ Software (Waters®) was used for data acquisition and processing.

### Effect of temperature, pH and oxidation in the stability and antiviral activity of P3

The stability and antiviral activity of P3 were evaluated at different conditions of temperature, pH and oxidation. Briefly, P3 solutions at concentrations corresponding to the IC_50_ and IC_90_ were prepared in GM and were incubated at 25, 37, and 65°C for a period of 1 week, a 37°C for a period of a month and at 4°C for a period of 4 months. The antiviral activity of each peptide solution was then evaluated with a single-round viral infectivity assay using TZM-bl reporter cells and HIV-1 isolate 93AOHDC250, as described above.

The effect of pH on P3 was evaluated at pH ranging from 4 to 8. Briefly, IC_50_ peptide solutions were prepared in GM and the pH was adjusted using HCl (1M) or NaOH (1M). Each solution was then incubated at 37°C for 2, 8 and 24 hours and their antiviral activity was determined in a single-round viral infectivity assay using a luciferase reporter gene assay in TZM-bl cells as described above.

To test for oxidation by H_2_O_2_, P3 solutions at concentrations corresponding to the IC_50_ and IC_90_ were treated with H_2_O_2_ at biologic relevant concentrations (1.2μM and 5μM) [29]. The antiviral activity of the different solutions was determined in a single-round viral infectivity assay using a luciferase reporter gene assay in TZM-bl cells, as described above.

Controls for these experiments were the following: positive controls (cells plus virus), negative controls (cells plus complete growth medium); pH compound controls (cells plus P3 in different pH growth medium); H_2_O_2_ compound controls (cells plus P3 in H_2_O_2_, 1.2μM or 5μM); cell controls were also performed under the same incubation conditions. H_2_O_2_ and pH cytotoxicity were assessed in TZM-bl cells using alamarBlue assay [28]. In all cases, P3 was considered stable when its antiviral activity was unaffected under the different conditions tested.

### Hydroxyethyl cellulose P3 gel

Hydroxyethyl cellulose (HEC) powder (Ashland, Switzerland) was sterilized by dry heat at 150°C for 24h. The sterility was confirmed by direct inoculation of HEC powder (0.1mg) in Sabouraud agar, blood agar and trypticase soy agar. Blood and trypticase soy agar plates were incubated 24h at 37°C and Sabouraud agar plates 48h at 37°C. Aqueous systems containing decreasing concentrations (%) of HEC (10, 7.5, 5, 2.5, 1.5, 1, 0.5, 0.25 and 0.05) were prepared to assess the HEC-gel cytotoxicity in TZM-bl cells using the alamarBlue assay [28].

P3 (366.4 nM corresponding to the IC_90_) was formulated in 1.5% HEC-gel with 20% X-Gal (5-bromo-4-chloro-3-indolyl-beta-D-galacto-pyranoside). This concentration of HEC was chosen based in the results of the cytotoxicity assay and the feasibility of the in vitro antiviral assay.

The antiviral activity of this gel (designated P3/X-Gal HEC-gel) was evaluated using a single-round infectivity assay in TZM-bl reporter cells. Briefly, TZM-bl cells were plated (1x10^4^ cells/well) in a 96-plate and incubated overnight at 37°C with 5% CO_2_. In the following day, the P3/X-Gal HEC-gel was added to each well in a total volume of 100 μl. Cells were then infected with 200 TCID50 (in 100 μl) of HIV-1 isolate 93AOHDC250 and incubated for five days at 37°C with 5% CO_2_. The TZM-bl cell line has an integrated copy of the β-galactosidase gene under control of the HIV-1 promoter enabling simple and quantitative analysis of HIV infection using β-galactosidase as a reporter gene. When HIV infects TZM-bl cells, β-galactosidase gene is expressed and the X-gal present in the medium is hydrolyzed by the β-galactosidase enzyme producing an intensely blue product (5,5'-dibromo-4,4'-dichloro-indigo)[30]. The cells were observed directly in an inverted microscope coupled with a Leica DFC490 camera at 100X magnification. At least two independent experiments were performed, each in triplicate wells. Controls for these experiments were the following: positive control (cells + X-Gal HEC-gel + virus); negative control (cells + X-Gal HEC-gel); compound control (cells + P3/X-Gal HEC-gel); all of the above controls without X-Gal.

### Spermicidal activity

Sperm was obtained from normal donors (n = 3) after signing a written informed consent. The sperm was incubated at 37°C with a 114 μM P3 solution in water (40%) and PBS (60%) (≈300-fold higher concentration than the IC_90_) and sperm motility and viability were analyzed at various time intervals (0, 30, 60, 120 and 240 min). Spermatozoa viability was evaluated by dye exclusion method using a solution of Eosin Y (5mg/mL). Two hundred spermatozoa were counted with a phase-contrast microscope (Olympus, Modell BH-2), differentiating the live (unstained) spermatozoa from the dead (stained) cells. Sperm motility was evaluated in a Makler chamber using a computer-aided sperm analysis system (CASA, Hamilton Thorne Research, MA, USA). At least five microscopic fields were assessed in a systematic way to classify 200 spermatozoa motility. Each spermatozoon is graded according to cell velocity: progressive motility (i.e.,>5 μm/s), non-progressive motility (<5 μm/s) and immotility [31].

### Anti-bacterial activity

Anti-bacterial activity of P3 was determined according to CLSI guidelines [32]. The following bacteria were used: reference strains *E*. *coli* ATCC 10536, *Staphylococcus aureus* ATCC 6538, *Bacillus subtilis* ATCC 6633 and *Enterococcus faecalis* ATCC 29212; clinical strains of *E*. *coli* and *Pseudomonas aeruginosa* isolated in our laboratory; and strains of *Lactobacillus rhamnosus* and *Lactobacillus plantarum* also isolated in our laboratory. The Minimum Inhibitory Concentration (MIC) was determined by the agar diffusion method, in plates of Mueller-Hinton agar or Rogosa agar (for Lactobacilli) [33]. Briefly, 10^8^cfu/ml bacterial suspensions were prepared in sterile water and spread in the culture media. Sterile disks containing different concentrations of P3 were placed on the inoculated surface. Plates were incubated at 37°C for 24h or 48h. Lactobacilli were incubated at microaerophilic conditions. A negative control made of sterile water was used. Disks of amoxicillin and imipenem were used as positive controls. The maximum concentration of P3 tested was 228.4 μM (≈600-fold higher concentration than the IC_90_).

### BALB/c mice vaginal irritation test

Female BALB/c mice (6–8 weeks-old) (Charles River Laboratories, Wilmington, MA) were pretreated subcutaneously with 2mg of medroxyprogesterone acetate (Depo-Provera; Pfizer, NY, USA) in PBS (Lonza, Verviers, Belgium). After five days, 30μl of test formulation with different doses of P3 diluted in PBS was applied intravaginally daily for 7 days by vaginal gavage to animals previously anesthetized with isoflurane (Forane, Abbott, Madrid). Controls included mice that received PBS (placebo group) and 3% nonoxynol-9 (N9) in PBS (irritation control group). On the eighth day, mice were sacrificed and genital tract tissue were extracted and fixed in 4% paraformaldehyde for histological analysis. The animals were distributed in groups of three mice each: group placebo only treated with PBS (1–3), group A treated with dose of 0.06mg/day (456.8 μM) of P3 in PBS (4–6), group B treated with dose of 0.2mg/day (1522.7 μM) of P3 in PBS (7–9), group C treated with dose of 0.4mg/day (3045.3 μM) of P3 in PBS (10–12) and group irritation control treated with 3% N9 in PBS (13–15).

### Histological studies in BALB/c

The presence of histological lesions in the mouse vaginas was evaluated with hematoxylin-eosin staining. Samples were embedded in paraffin by submersion in increasing concentrations of ethanol in water (Rectapur, VWR, England), two baths of xylene (Analar (VWR, England) and another of paraffin, before being placed in a paraffin mold. Subsequently, they were cut using a semimotorized microtome (RM2145 Leica, Germany) and processed for staining. For dewaxing, samples were submersed into two baths of xylene solution (10 min) and three baths in descending order of ethanol (100%, 90% and 70%) (5 min), before being stained with hematoxylin (Merck, Germany) for 5 min and eosin (Merck, Germany) for another 5 min. Post-eosin staining dehydration was performed with passages in increasing concentrations of ethanol (70%, 90% and 100%) and a bath of xylene solution. Finally, they were mounted with D.P.X. (Prolabo, VWR, Spain).

The existence of injury in vaginal epithelium, inflammatory infiltrate, vascular congestion and/or edema in the submucosa was evaluated in each biological sample. The values (score) assigned for each of these lesions were: 0 (no change) when no injury or the observed changes were within normal range; 1 (minimum) when changes were sparse but exceeded those considered normal; 2 (light) when injuries were identifiable but with no severity; 3 (moderate) for significant injury that could increase in severity; 4 (very serious) for very serious injuries that occupy most of the analyzed tissue. These values were added up and determined the level of vaginal irritation as minimum 1–4, average 5–8, moderate 9–11 and severe 12–16 [34].

### Statistical analysis

Results of stability studies were analyzed by one-way ANOVA (Friedman test) with a post-test of multiple comparisons (Dunn´s test). Sperm motility and viability statistical data analysis was made using the one-way ANOVA with a post-test of multiple comparisons (Sidak´s correction) between the drug vehicle group (control) and the P3 group, for each incubation time (significance at p<0.05).

### Ethic statement

The study was approved by the ethics committees of the participating institutions. The methods were performed in accordance with the relevant guidelines and regulations. All experiments involving human participants were performed according to the guidelines and protocols approved by the Ethics Committee of Faculdade de Farmácia da Universidade de Lisboa. All animal experiments were performed according to the guidelines and protocols approved by the Ethics Committee for Animal Experimentation of Comunidad Autónoma de Madrid, CAM, Spain. Oral informed consent was obtained from all subjects enrolled in this study.

## Results

### Antiviral activity of P3 is unaffected in the presence of seminal plasma and vaginal fluid simulant

**Fig 1:**
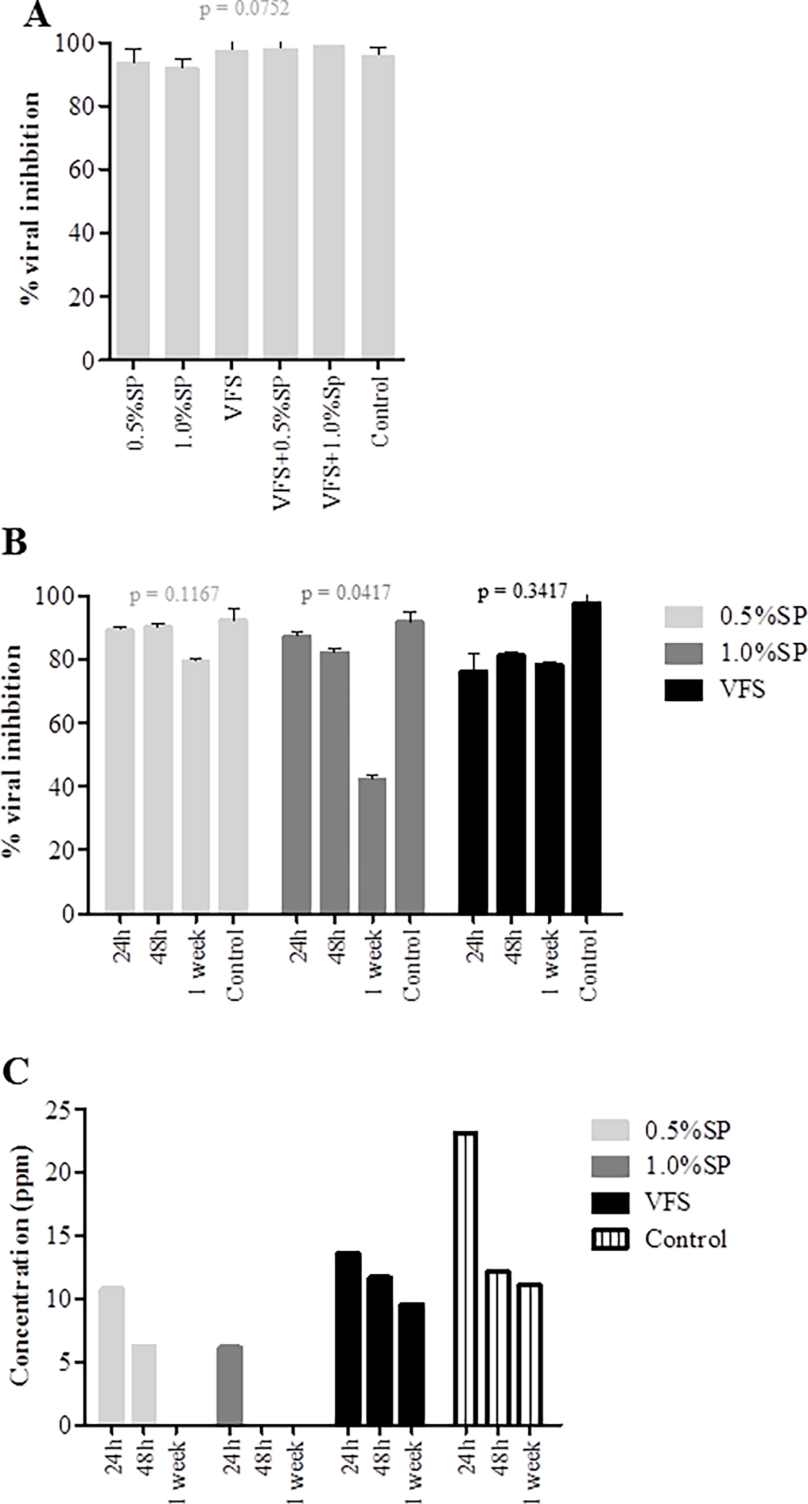
**Evaluation of P3 anti-HIV1 activity in the presence of genital fluids: (A) SP, VFS and the combination of SP and VFS, (B) SP and VFS in different time points. (C) Effect of SP and VFS on P3 as determined by mass spectrometry.** Solutions of P3 (366.4nM) were prepared in complete growth medium in the presence of 0.5% or 1% (v/v) SP, VFS, VFS with 0.5% or 1% (v/v) SP. The antiviral activity of each solution was evaluated with a single-round viral infectivity assay using TZM-bl reporter cells and HIV-1 primary isolate 93AOHDC250 in the presence of growth medium alone, SP and VFS to define the effects of biologic fluids on antiviral activity.

The anti-HIV activity of a microbicide candidate should be tested in the presence of the biological fluids encountered during the sexual transmission event [35]. Thus, the antiviral activity of P3 was evaluated in the presence of seminal plasma (SP), vaginal fluid simulant (VFS) and the combination of both. We first confirmed that SP and VFS were not cytotoxic for TZM-bl cells at the concentrations used in these assays (0.5 and 1.0%) (S1 Fig). The antiviral activity of P3 was unaffected by the human fluids up to 1 week of contact (Fig 1). The only exception was SP 1% that caused a significant decrease of antiviral activity at 1 week. Consistent with the virological results, P3 was readily detectable in the presence of VFS by mass spectrometry. However, there was a decrease to undetectable levels of P3 [below the limit of quantification of the method which is 2.5μg/mL (2.5 ppm)] after one week in the presence of 0.5% of SP and after 48h in the presence of 1% SP (Fig 1C). This indicates that P3 is degraded to some extent in the presence of SP and explains the significantly lower antiviral activity of P3 after one week with SP. Overall these results indicate that P3 maintain full antiviral activity in the presence of human biological fluids during at least 48h.

### Antiviral activity of P3 is preserved at stringent temperature, pH and oxidative stress conditions

**Fig 2:**
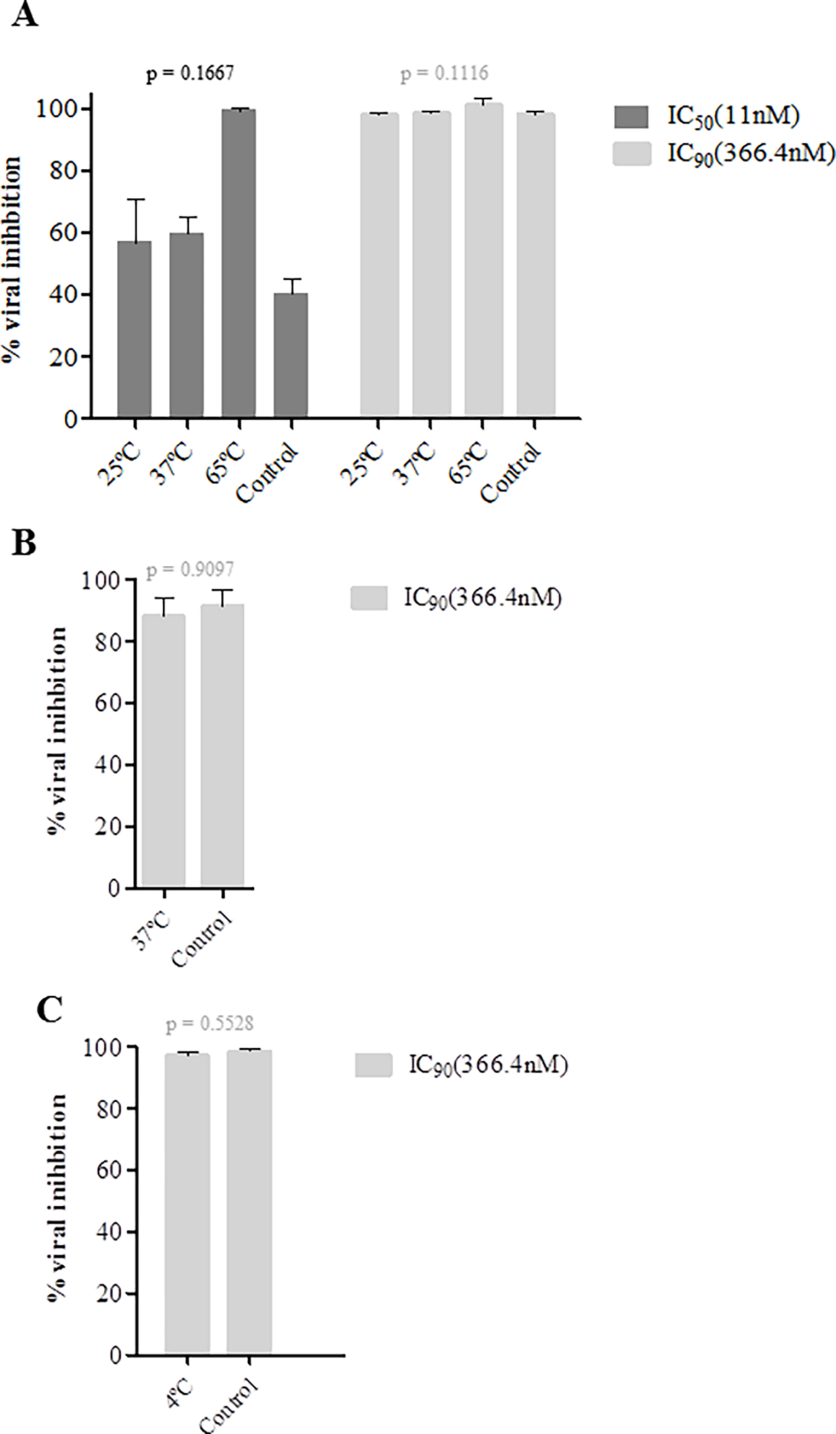
Biological stability of P3 at different temperatures. The stability of P3 solutions (11 and/or 366.4nM) in growth medium were evaluated in a biological assay as a function of temperature. Thermal degradation studies were conducted at 25, 37, and 65°C for a period of 1 week (A), 37°C for a period of a month (B) and a 4°C for a period of 4 months (C). The antiviral activity of each solution was evaluated with a single-round viral infectivity assay using TZM-bl reporter cells and HIV-1 primary isolate 93AOHDC250.

The P3 peptide must remain stable at different temperatures during the manufacturing process of the microbicide, during the shelf-life of the compound and in the human body [36]. Since the microbicide will be applied vaginally P3 must be stable in the acidic environment of the healthy vagina (pH 3.5–4.5) [37] and in the near neutral pH environment after ejaculation [38]. Finally, it should not be oxidized by the H_2_O_2_ present in the vaginal lumen [39, 40]. To test for thermal stability, P3 concentrations corresponding to the IC_50_ (11nM) and IC_90_ (366.4nM) were incubated at 25°C, 37°C and 65°C for a period of one week, a 37°C for a month and at 4°C during 4 months and then its antiviral activity was determined and compared to the control in which antiviral activity was determined after 48h at 37°C. Similar mean viral inhibition levels were obtained after a week incubation at 25°C (IC50, 57.5% vs control 43.5% p = 0.3338 and IC90, 99% vs control 99% p = 0.5, Friedman Test) and at 37°C (59.5% vs 43.5% p = 0.3366 and 99% vs 99% p = 0.5) (Fig 2A). Although not statistically significant, antiviral activity of P3 increased at 65°C (99.5% vs 43.5% p = 0.3301 and 100% vs 99% p = 0.5). P3 stored at 37°C for a month and stored at 4°C during four months maintained full anti-HIV-1 activity (Fig 2B and 2C). Together, these results highlight the very high thermal stability of P3.

**Fig 3:**
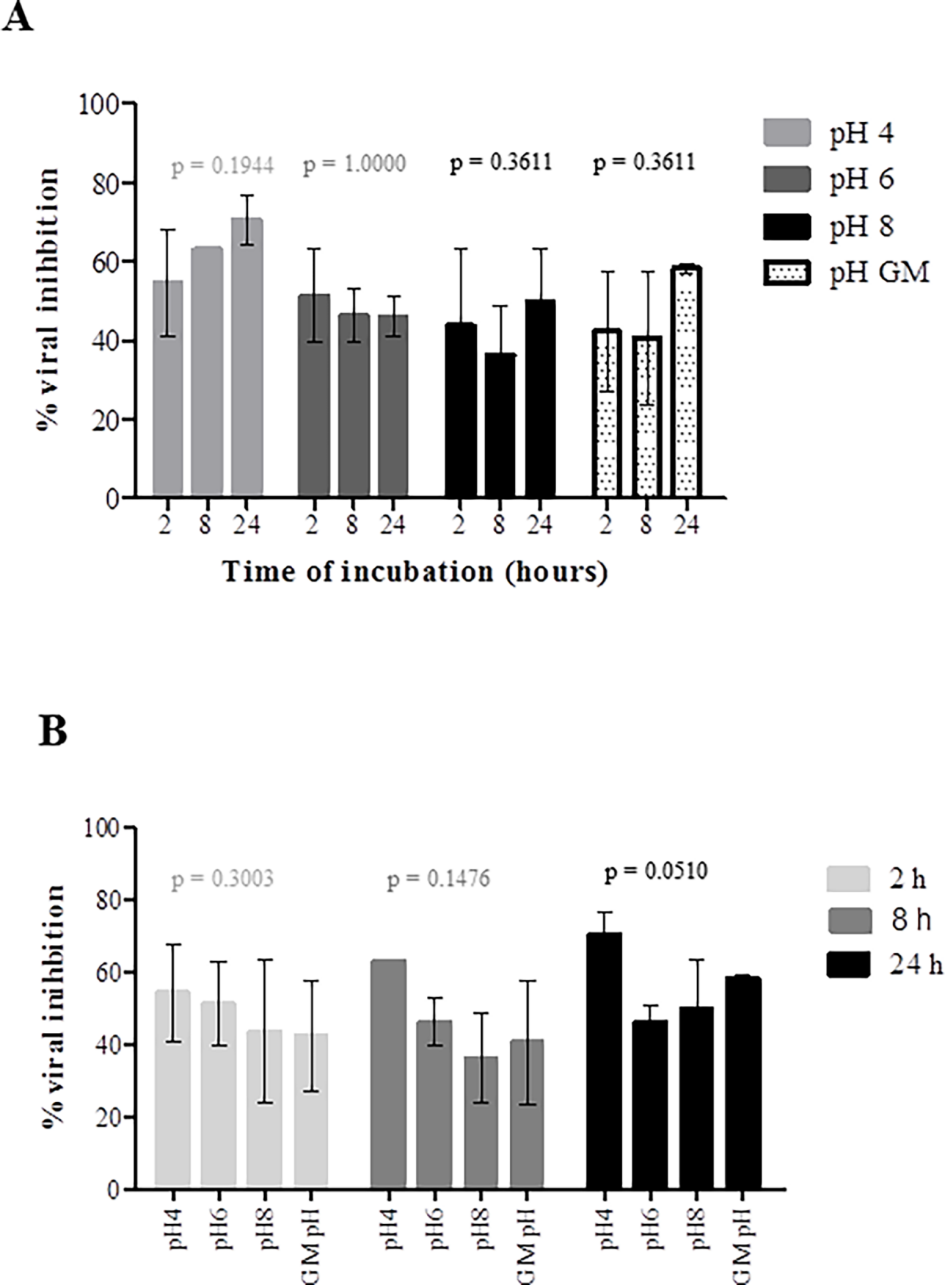
Biological stability of P3 at different pH conditions. 11nM solutions of P3 (corresponding to the IC_50_) in growth medium (GM) at pH 4, 6 and 8 were incubated at 37°C for 2, 8, 24 hours. The antiviral activity of each solution was evaluated with a single-round viral infectivity assay using TZM-bl reporter cells and HIV-1 primary isolate 93AOHDC250.

**Fig 4:**
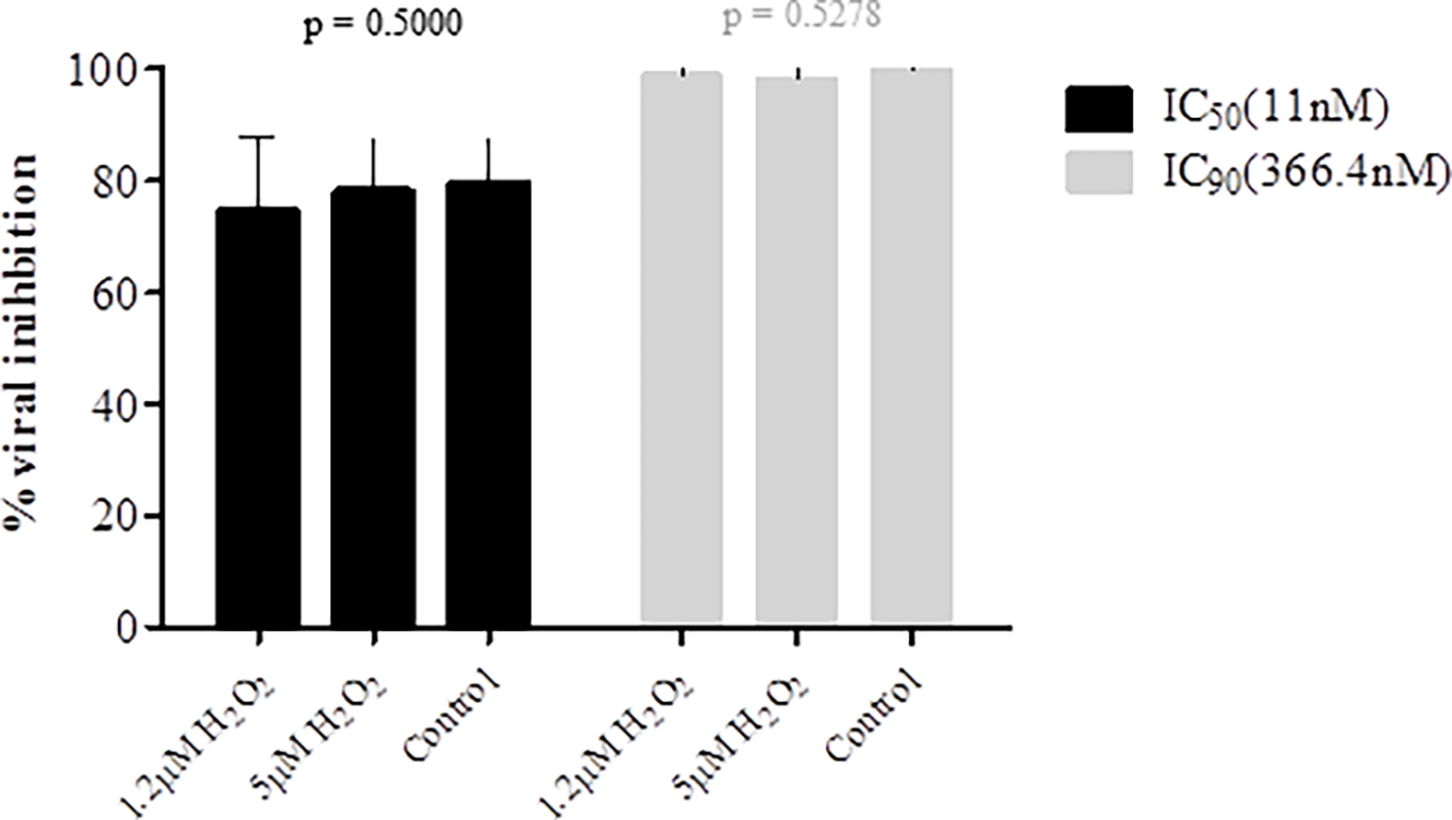
Antiviral activity of P3 in the presence of H_2_O_2_. Solutions of P3 (11 and 366.4nM) were prepared in complete growth medium in the presence of 1.2μM of H_2_O_2_ and 5μM of H_2_O_2_. The antiviral activity of each solution was evaluated with a single-round viral infectivity assay using TZM-bl reporter cells and HIV-1 primary isolate 93AOHDC250.

Next we accessed the stability of P3 at different pH (4, 6, 8) and for different periods of time (2h, 8h and 24h). For these experiments a concentration equivalent to the IC_50_ was used. TZM-bl cells cytotoxicity was first analyzed. Basic P3 solution (pH = 8) did not affect the cells; acidic solutions (pH = 4 and 6) affected only minimally cell viability (S2 Fig). For each pH condition tested, P3 always inhibited around 50% viral replication indicating that the pH did not significantly affect its antiviral activity (after 2h incubation at pH = 4, mean = 56.67±9.504%; pH = 6, 51.33±11.68%; pH = 8, 43.67±19.66%; pH = 7.5 GM (control), 42.33±15.31%; p = 0.3004; after 8h incubation- pH4, 62.67%±0.5774; pH6, 46.33%±6.658; pH8, 36.33%±12.420; pH7.5 GM (control), 40.67%±17.01; p = 0.1476; 24h- pH4, 70.67%±4.509; pH6, 46.00%±5.000; pH8, 49.67%±13.650; pH7.7 GM (control), 58.33%±1.155; p = 0.0510) (Fig 3). P3 activity seemed to be potentiated at pH = 4 after 24h (Fig 3B) but no significant differences were found when compared with the other pH conditions and with the control (P3 in GM, pH = 7.5). Finally, P3 was stable and maintained its antiviral activity in the presence of biological concentrations of H_2_O_2_ (1.2μM and 5μM) (Fig 4). H_2_O_2_ at these concentrations did not affect the viability of TZM-bl cells (S3 Fig). Taken together, the high stability of P3 at different temperature, pH and oxidative conditions indicate that it is an ideal candidate for a vaginal microbicide.

### P3 exerts full antiviral activity in the context of the HEC-gel

**Fig 5:**
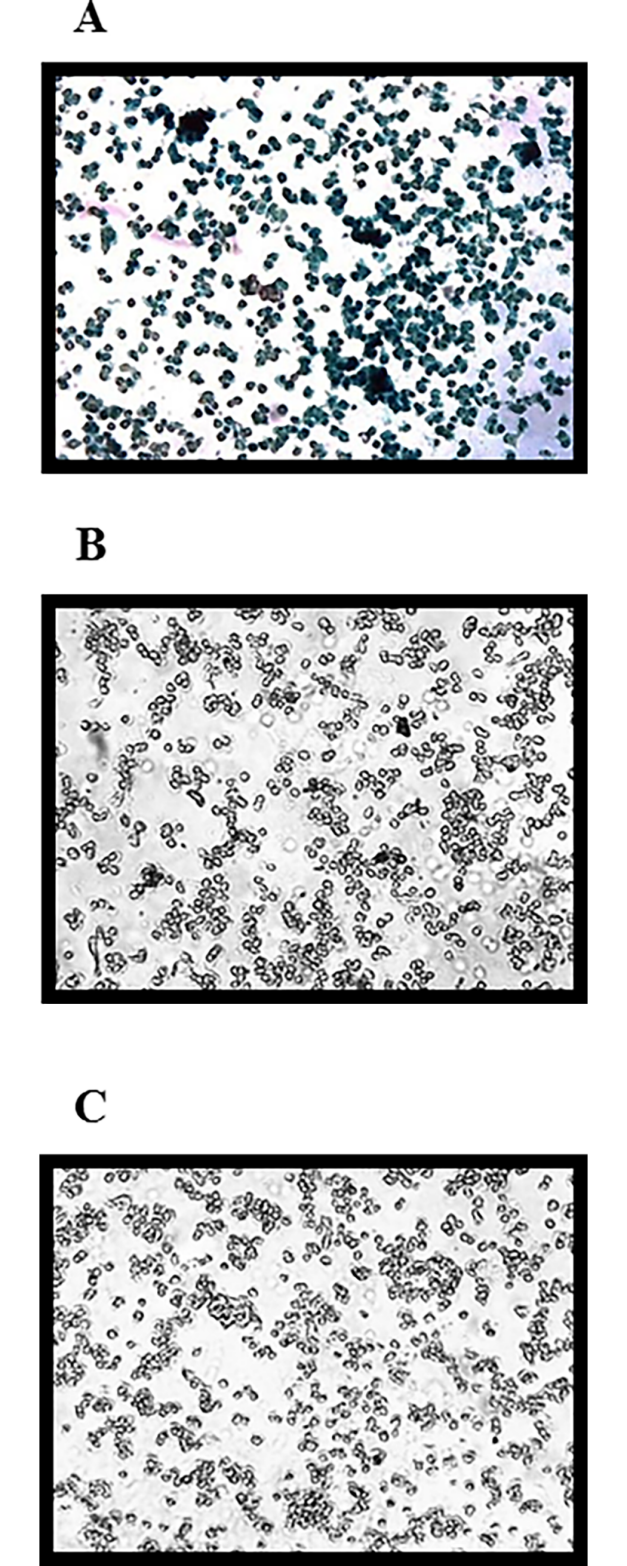
Antiviral activity of P3 formulated in 1.5% HEC-gel in TZM-bl cells. (A) 1.5% HEC-gel + X-Gal + HIV-1 (positive control); (B) 1.5% of HEC-gel + X-Gal (negative control); (C) 1.5% HEC-gel + P3 (366.4 nM) **+** HIV-1 + X-Gal. HIV infected cells produce β-galactosidase which converts X-Gal into 5,5'-dibromo-4,4'-dichloro-indigo, an intensely blue product which is insoluble. The cells were observed in an inverted microscope coupled with Leica DFC490 camera at 100X magnification.

As the aim of the research was to develop a P3-based HEC gel it was important to assess whether the HEC-gel interferes with the stability and antiviral activity of P3. We first determined that HEC-gel at concentration ≤1.5% is not cytotoxic to TZM-bl cells (S4 Fig). We therefore formulated P3 peptide at 366.4 nM concentration (equivalent to the IC_90_) in 1.5% HEC-gel. This gel showed full activity against HIV-1 thereby confirming that the P3 peptide is effectively delivered in the HEC-gel formulation (Fig 5).

### P3 lacks spermicidal activity

**Fig 6:**
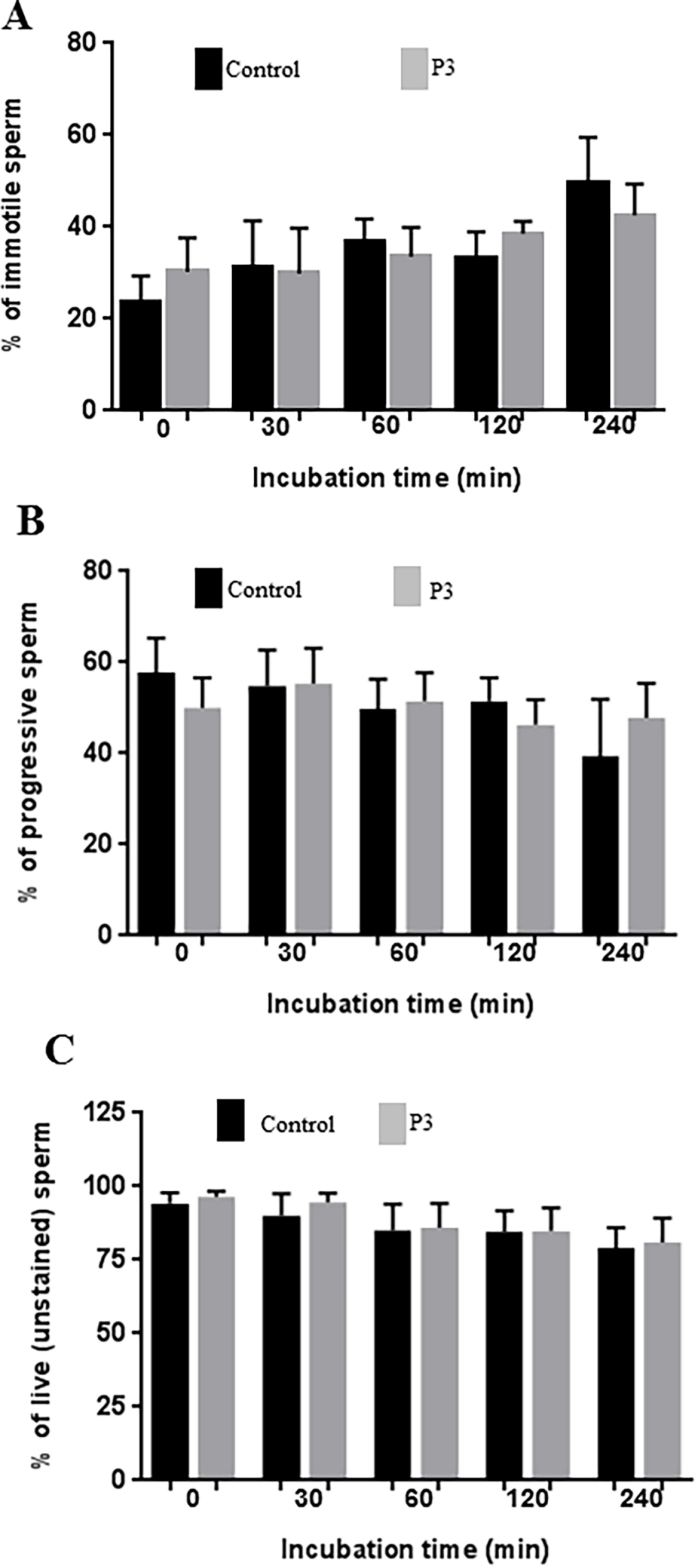
Effect of P3 in human spermatozoa motility and viability. Motility and vitality were assessed at different time intervals (0, 30, 60, 120 and 240 min). (A) Percentage of immotile sperm in the presence or absence (control) of P3; (B) Percentage of progressive sperm in the presence or absence (control) of P3; (C) Percentage of live sperm in the presence or absence (control) of P3. No differences (p>0.05) were observed between control group (drug vehicle) and P3 treated group, for each incubation time.

As a vaginal microbicide P3 will be in contact with semen and it is crucial that it does not alter the sperm characteristics. To assess the effect of P3 peptide in sperm cells, the semen was cultured in the presence of 114 μM of P3 (≈300-fold higher than the IC_90_) and the progressive motility and vitality of the sperm cells was analyzed at 0, 30, 60, 120 and 240 min post-treatment. No significant changes were found in spermatozoa motility and vitality (Fig 6) when compared with control without P3. These findings indicate that P3 lacks spermicidal activity and could be safely used as a vaginal microbicide.

### P3 lacks anti-bacterial activity

*Lactobacillus* species and other bacterial species that are present in a healthy vagina crucially contribute to maintain the 3.5 to 4.5 acidic pH and produce several antiviral and antimicrobial substances that inhibit pathogenic organisms [41]. Alterations of normal vaginal microbiota may lead to several vaginal infections [42] and affect the risk for vaginal HIV transmission [35, 43]. Hence, P3 must not interfere with the normal vaginal microbiota. The effect of P3 on vaginal bacteria was investigated using different species of bacteria including two common clinical species of lactobacillus, *Lactobacillus rhamnosus* and *Lactobacillus plantarum* (Table 1). P3 did not affect bacteria growth up to 228.4 μM concentration (≈600-fold higher than the IC_90_) (Table 1). These findings suggest that P3 will not affect the normal vaginal microbiota.

**Table 1:** Activity of the P3 peptide against gram positive and gram negative bacteria.

Bacteria	P3 (MIC^a^)
*Bacillus subtilis* ATCC 6633	> 228.4μM
*E*. *coli* ATCC 10536	> 228.4μM
*E*. *coli* (clinical strain)	> 228.4μM
*Enterococcus faecalis* ATCC 29212	> 228.4μM
*Lactobacillus plantarum*	> 228.4μM
*Lactobacillus rhamnosus*	> 228.4μM
*Pseudomonas aeruginosa* (clinical strain)	> 228.4μM
*Staphylococcus aureus* ATCC 6538	> 228.4μM

^a^Minimum Inhibitory Concentration at 228.4μM which is ≈600-fold higher than the IC_90._

### P3 does not significantly irritate or damage the vaginal mucosa

To see if P3 exposure induced alterations on the vaginal mucosa, histological evaluation of lesions found in the vaginal wall of 15 BALB-c mice treated with PBS, with P3 at doses of 0.06 mg, 0.2 mg and 0.4 mg (in 30μl) in PBS (456.8 μM, 1522.7 μM and 3045.3 μM, respectively) and with 3% N9 in PBS daily for 7 consecutive days, was done. In placebo group treated with PBS no significant histological lesions were observed in any of three samples tested (S5A–S5D Fig). The epithelium was composed of several layers of cells, being the last one, a layer of mucous cells that was present in the proestrus phase of the estrous cycle. No inflammatory infiltrate or vascular congestion was found in any layers of the wall.

In mice treated with 3% N9 considered as mucosal irritation control group (S5E–S5H Fig), no epithelial mucosal cell layer was found, but that it had been replaced by a stratified epithelium such as is present in estrus. Hyperplasia of the epithelium was observed, with a higher number of cell layers than normal condition, with evidence of papillary formations into the submucosa (S5E–S5H Fig). There was also an inflammatory infiltrate constituted mainly by polymorphonuclear neutrophils between epithelial cells which sometimes form microabscesses (S5H Fig). Focal ulceration also appears in some areas of tissue. This inflammatory infiltrate has spread to the submucosa with a moderate degree of severity. In this submucosa, light congestion and vasodilation were also observed (S5E–S5H Fig). The total score obtained in these three samples indicate the presence of an average irritation of the vaginal wall (Table 2).

**Table 2:** Histological score after intravaginal administration of compounds in different groups of female Balb/c mice during 7 consecutive days.

	PBS			456 μM/day P3			1522.7 μM/day P3			3045.3 μM/day P3			3% N9 P3		
Mouse ID	1	2	3	4	5	6	7	8	9	10	11	12	13	14	15
**Epithelial lesion**	0	0	0	1	1	0	1	0	0	4	2	4	3	2	3
**Inflammatory infiltrate**	0	0	0	0	1	0	1	0	2	3	0	2	3	3	3
**Vascular congestion**	0	0	0	1	0	0	1	1	2	3	1	2	2	2	2
**Edema/fibrosis**	0	0	0	0	1	0	0	0	0	0	0	1	0	0	0
Total Score*	0	0	0	2	3	0	3	1	4	10	3	9	8	7	8

*The values (score) assigned for each of these lesions were: 0 (no change) when no injury or the observed changes were within normal range; 1 (minimum) when changes were sparse but exceeded those considered normal; 2 (light) when injuries were identifiable but with no severity; 3 (moderate) for significant injury that could increase in severity; 4 (very serious) for very serious injuries that occupy most of the analyzed tissue. These values were added up and determined the level of vaginal irritation as minimum 1–4, average 5–8, moderate 9–11 and severe 12–16.

In group A treated with 0.06 mg/day (456.8 μM) of the compound, injuries were minimal or even absent (mice 6), histological alterations were not showed (S6 Fig). As in the case of the samples treated only with PBS, mucosal epithelial cells were maintained and only minimal epithelial hyperplasia without presence of inflammatory cells appeared. In sample 4 scarce congestion and vasodilatation were observed and the sample 5 showed a minimal infiltration of neutrophils in the submucosa.

In group B (S7 Fig) treated with 0.2 mg/day (1522.7 μM), lesions were less obvious than in mice treated with doses of 0.4 mg/day (3045.3 μM). Only in the sample 7 appeared minimal inflammatory infiltrate in the epithelium, like in the beginning of the metaestrus at the estrous cycle. This inflammation was extended into the submucosa, being minimal in this layer the vascular congestion and vasodilation S7A and S7B Fig). In the sample 9, the inflammatory infiltrate and congestion were light (S7E and S7F Fig). In this group the irritation of the vaginal wall was minimal.

Finally, in group C treated with 0.4 mg/day (3045.3 μM) of P3 peptide, two of the samples (mice 10 and 12) showed the most obvious lesions of the groups studied. The epithelium kept mucosal cells own of proestrus, but many neutrophils were observed inducing microabscesses. In addition, hyperplasia remains and numerous apoptotic cells appeared. The injuries were severe. In the submucosa, an inflammatory infiltrate consistent of neutrophils was found and a moderate level of vasodilation and congestion occurred (S8A, S8B, S8E and S8F Fig). Score of vaginal irritation was moderate in both cases. However, sample 11 had only a slight epithelial hyperplasia and minimal vasodilatation in the submucosa (S8C and S8D Fig) (Table 2). Overall, the most severe injuries were found in two mice belonging to the group treated with 0.4 mg/day (3045.3 μM), with moderate vaginal irritation. However, in another mouse of that same group, the irritation of the vaginal epithelium was minimal. The samples belonging to mice treated with 3% N9 showed an average degree of vaginal irritation. In the rest of mice, treated groups and placebo group, mild histological lesions were observed in their samples. Taken together these results indicate that P3 could be safely administered in a microbicide at a dose up to 0.2 mg/day (1522.7 μM).

## Discussion

There is an urgent need for female-controlled HIV prevention strategies. A safe and effective vaccine against HIV would be the best solution but despite more than two decades of HIV-1 vaccine research there is still no efficacious vaccine. The use of vaginal microbicides is one strategy to provide women with the ability to prevent HIV transmission from their infected partners. However, the most promising microbicides tested up to now, which contain TDF or dapivirine as active drugs, have conferred only modest protection against HIV-1 acquisition in women [1, 16, 18]. Concerns are also raised regarding the efficacy of TDF-based microbicides against infection by TDF-resistant strains, which are now common in low- and middle-income regions, and possible selection of TDF-resistant strains which will not respond to a TDF-based treatment regimen [11, 20].

We have recently described a fusion inhibitor peptide, named P3, with very potent activity against HIV-1, HIV-2 and SIV [22, 44]. Potent and specific entry inhibitors like P3 that are not in clinical use may be interesting microbicide candidates for HIV prevention. In this work we evaluated the activity of the P3 peptide in a gel of hydroxyethyl cellulose (HEC) using a newly developed *in vitro* assay. HEC has been used as the universal placebo gel in several vaginal gel formulations and is considered safe [45, 46]. We found that P3 at the IC_90_ concentration was effectively delivered to the target cells in a 1.5% HEC-gel and was fully active against HIV-1.

A microbicide candidate must remain stable in the presence of genital fluids, sperm and vaginal fluid, which will be present in the sexual act. The healthy human vaginal environment is acidic (pH 3.5–4.5) and contains proteolytic enzymes that may inactivate anti-HIV microbicides. Similarly the antiviral activity of polyanionics compounds and several candidate gels (cellulose sulfate and PRO 2000) was negatively affected by the presence of semen/seminal plasma (SP) [13–15, 47–49]. We investigated the stability and bioactivity of P3 in the presence of SP and vaginal fluid simulant (VFS) and found that its anti-HIV-1 activity was not affected in the presence of these human fluids, even after a month of exposition. P3 peptide also remained stable in a pH range of 4–8, even after 24 hours incubation. Remarkably, at pH = 4 which is the pH of the healthy vagina, the anti-HIV-1 activity of P3 was even better than at more alkaline pH. Bacterial vaginosis is a human vaginal infection in which the vaginal pH is increased towards alkaline values (pH>4.5) [50]; such pH changes may contribute for spontaneous hydrolysis and degradation of anti-HIV compounds [51]. In our case the antiviral activity of P3 was not affected at pH 6 and 8. Taken together, these results suggests that the antiviral activity of P3 should not be affected in the vaginal lumen even after intercourse, and that it could also be active in the setting of bacterial vaginosis.

Microbicides must be stable at 37°C for a long period of time since it is the body temperature, and at 65ºC since some manufacturing processes may require high temperatures [40]. Stability at room temperature or at 4ºC is also important since the storage at these temperatures is easier for the user and more affordable than at -20ºC. No statistically significance differences were observed on the anti-HIV-1 activity of P3 peptide stored at 25, 37 and 65ºC for a period of a week. The stability of P3 under different temperatures is much better than other peptide-based microbicide candidates or drugs; for example, retrocyclin RC-101 is susceptible to degradation at 65º C [40] and Spantide II is not stable at temperatures higher than 40ºC [52]. The stability of P3 under different temperatures makes it an ideal microbicide to be used in places with high ambient temperature and with limited access to cold storage chains.

Vaginal microbicide products will be in contact with the healthy vaginal microbiota, especially with *Lactobacillus* species that produce hydrogen peroxide (H_2_O_2_) which could oxidize P3 [42]. The effect of H_2_O_2_ at biologically relevant concentrations on P3 was assess and it was found that it maintained full antiviral activity suggesting that it will not be oxidized in the healthy vaginal environment.

Vaginal microbicides should not cause lesions to the vaginal epithelium, which are associated with increased rates of HIV-1 acquisition, and should not be spermicidal [15, 53–56]. The P3 peptide neither altered the sperm motility or vitality nor caused significant alterations of the vaginal epithelium or vaginal irritation in Balb/C mice at 0.06 (456.8 μM) and 0.2 mg/day (1522.7 μM). Major injuries to the vaginal epithelium were found only in two mice treated with 0.4 mg/day (3045.3 μM) of P3 a dose which is ≈8300-fold higher than the IC_90_. Finally, P3 did not affect bacteria growth up to a concentration of 228.4 μM (≈600-fold higher than the IC_90_). This is important because a vaginal microbicide should preserve the normal vaginal microbiota, especially the *Lactobacillus* species that crucially contribute to the health of the vagina by producing lactic acid [57, 58]. Collectively, these findings indicate that P3 can safely be used as a vaginal microbicide.

In conclusion, the high anti-HIV potency of P3 in a HEC-gel, its high stability in genital fluids and in a wide range of pH and temperatures and in the presence of hydrogen peroxide, as well as the low or null cellular and bacterial toxicity makes P3 an excellent candidate for the development of a vaginal microbicide gel for the prevention of HIV transmission in women.

## Supporting information

S1 Fig Cellular viability of TZM-bl cells in the presence of biologic fluids. Cells were cultured in the presence of serial-fold dilutions of the biologic fluid and cellular viability was investigated using the alamarBlue assay. The grey dotted line corresponds to the concentration of VFS used in the assays and the black ones to SP concentrations.
(PDF)

S2 Fig Cellular viability of TZM-bl cells in the presence of different pH solutions. Cells were cultured in the presence of serial-fold dilutions of growth medium solutions at different pHs and cellular viability was investigated using the alamarBlue assay. The dotted line corresponds to the concentration of P3 pH solution used in the assays.
(PDF)

S3 Fig Cellular viability of TZM-bl cells in the presence of different concentrations of H_2_O_2_. Cells were cultured in the presence of serial-fold dilutions of H_2_O_2_ solutions and cellular viability was investigated using the alamarBlue assay. The dotted lines correspond to the concentration of H_2_O_2_ used in the assays.
(PDF)

S4 Fig Cellular viability of TZM-bl cells in the presence of different concentrations of HEC-gel. Cells were cultured in the presence of serial-fold dilutions of HEC-gel and cellular viability was investigated using the alamarBlue assay. The dotted line corresponds to the concentration of HEC-gel used in the assays (1.5%).
(PDF)

S5 Fig Immunohistochemical analyses of vaginal epithelium in Balb/c mice of control groups after intravaginal administration of PBS (placebo group) or 3% N9 (irritation control group) during 7 consecutive days. A, B: PBS-mice 1; C, D:PBS-mice 3. In neither case histological lesions were observed. E, F: mice 13 treated with 3% N9. Signs of epithelial hyperplasia and inflammatory infiltrate in the submucosa and congestion (arrows) were found. G, H: mice 15 treated with 3% N9 showed hyperplasia and presence of inflammatory cells in the mucosal epithelium (arrows).
(PDF)

S6 Fig Immunohistochemical analyses of vaginal epithelium in Balb/c mice after intravaginal administration of 0.06mg/day (456.8 μM) of P3 (A group) during 7 consecutive days. A, B: mice 4 showed minimal epithelial hyperplasia and vascular congestion; C, D: mice 5 presented epithelial hyperplasia and a slight inflammatory infiltrate in the submucosa (arrow). E, F: mice 6 without significant histological lesions.
(PDF)

S7 Fig Immunohistochemical analyses of vaginal epithelium in Balb/c mice after intravaginal administration of 0.2mg/day (1522.7 μM) of P3 (B group) during 7 consecutive days. A, B: mice 7 showed minimal inflammatory infiltrate in the epithelium and limited congestion in the submucosa; C, D: mice 8 presented scarce congestion in submucosa; E, F: mice 9 showed minimal inflammatory infiltrate and congestion in the submucosa.
(PDF)

S8 Fig Immunohistochemical analyses of vaginal epithelium in Balb/c mice after intravaginal administration of 0.4mg/day (3045.3 μM) of P3 (C group) during 7 consecutive days. A, B: mice 10 showed severe injury in the epithelium with hyperplasia and inflammatory infiltrate, as well as moderate congestion and inflammatory infiltrate in the submucosa; C, D: mice 11 showed minimal vascular congestion and minimal hyperplasia in the epithelium. E, F: mice 12 presented severe lesions in epithelium with inflammatory infiltrates (arrows) extending throughout submucosa although less evident here.
(PDF)
