# Correction: Evaluation of the fusion inhibitor P3 peptide as a potential microbicide to prevent HIV transmission in women

**DOI:** 10.1371/journal.pone.0197015

**Published:** 2018-05-02

**Authors:** Inês Bártolo, Ana Rita Diniz, Pedro Borrego, João Pedro Ferreira, Maria Rosário Bronze, Helena Barroso, Rui Pinto, Carlos Cardoso, João F. Pinto, Rafael Ceña Diaz, Pilar Garcia Broncano, Maria Angel Muñoz-Fernández, Nuno Taveira

An affiliation for the thirteenth author is missing. Nuno Taveira is also affiliated with #3 Centro de Investigação Interdisciplinar Egas Moniz (CiiEM), Instituto Superior de Ciências da Saúde Egas Moniz, Caparica, Portugal.
