# Supplementary figures and images for: Evaluation of the fusion inhibitor P3 peptide as a potential microbicide to prevent HIV transmission in women
Source: PLoS One. 2018 Apr 18;13(4):e0195744. doi: 10.1371/journal.pone.0195744 (PMC5905968; doi:10.1371/journal.pone.0195744)

S1 Fig

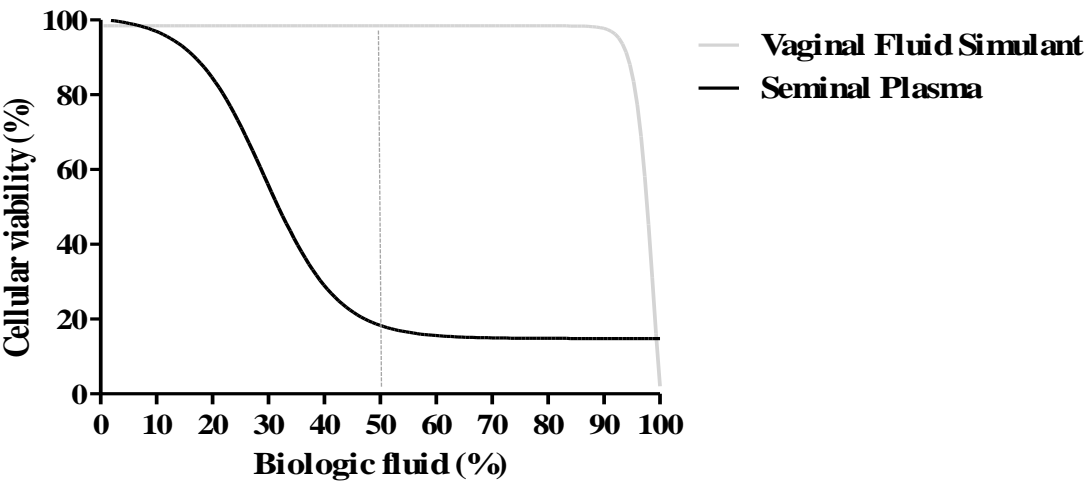

Supplement: S1 Fig — Cells were cultured in the presence of serial-fold dilutions of the biologic fluid and cellular viability was investigated using the alamarBlue assay. The grey dotted line corresponds to the concentration of VFS used in the assays and the black ones to SP concentrations. (PDF) [file pone.0195744.s001.pdf]

S2 Fig

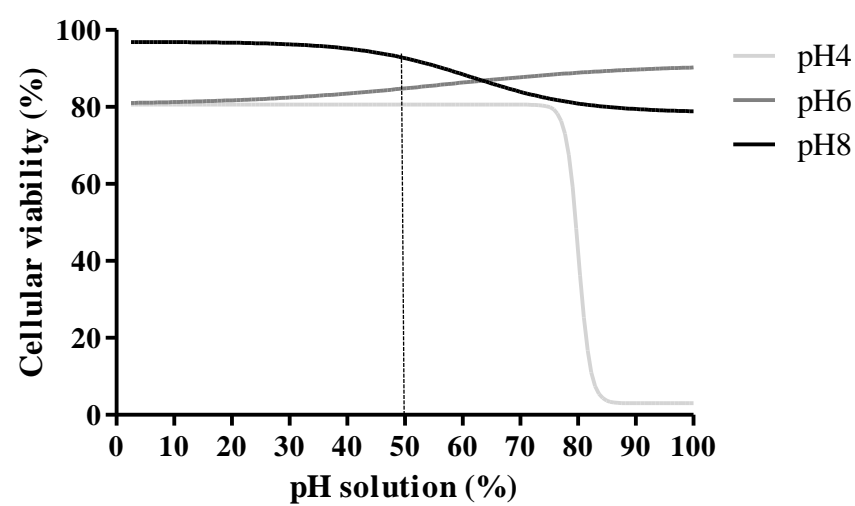

Supplement: S2 Fig — Cells were cultured in the presence of serial-fold dilutions of growth medium solutions at different pHs and cellular viability was investigated using the alamarBlue assay. The dotted line corresponds to the concentration of P3 pH solution used in the assays. (PDF) [file pone.0195744.s002.pdf]

**S3 Fig**

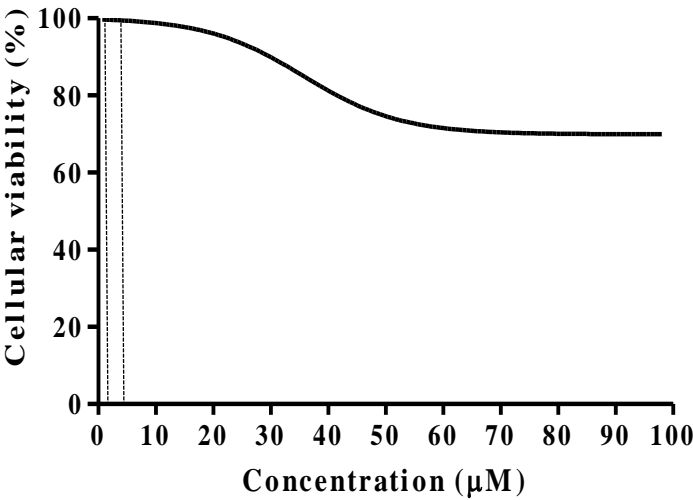

Supplement: S3 Fig — Cells were cultured in the presence of serial-fold dilutions of H2O2 solutions and cellular viability was investigated using the alamarBlue assay. The dotted lines correspond to the concentration of H2O2 used in the assays. (PDF) [file pone.0195744.s003.pdf]

**S4 Fig**

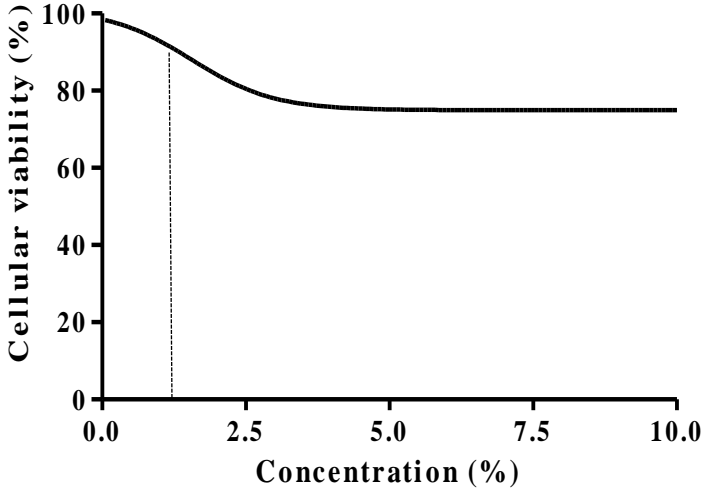

Supplement: S4 Fig — Cells were cultured in the presence of serial-fold dilutions of HEC-gel and cellular viability was investigated using the alamarBlue assay. The dotted line corresponds to the concentration of HEC-gel used in the assays (1.5%). (PDF) [file pone.0195744.s004.pdf]

**S5 Fig**

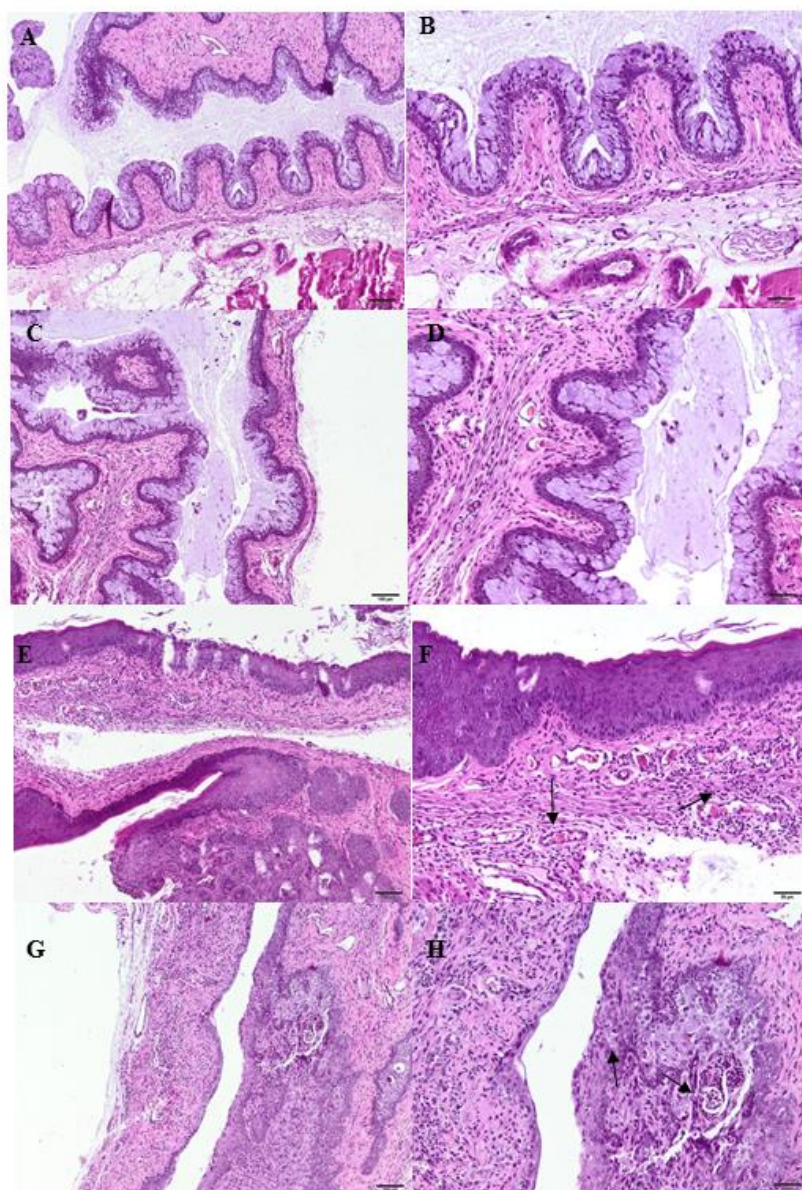

Supplement: S5 Fig — A, B: PBS-mice 1; C, D:PBS-mice 3. In neither case histological lesions were observed. E, F: mice 13 treated with 3% N9. Signs of epithelial hyperplasia and inflammatory infiltrate in the submucosa and congestion (arrows) were found. G, H: mice 15 treated with 3% N9 showed hyperplasia and presence of inflammatory cells in the mucosal epithelium (arrows). (PDF) [file pone.0195744.s005.pdf]

**S6 Fig**

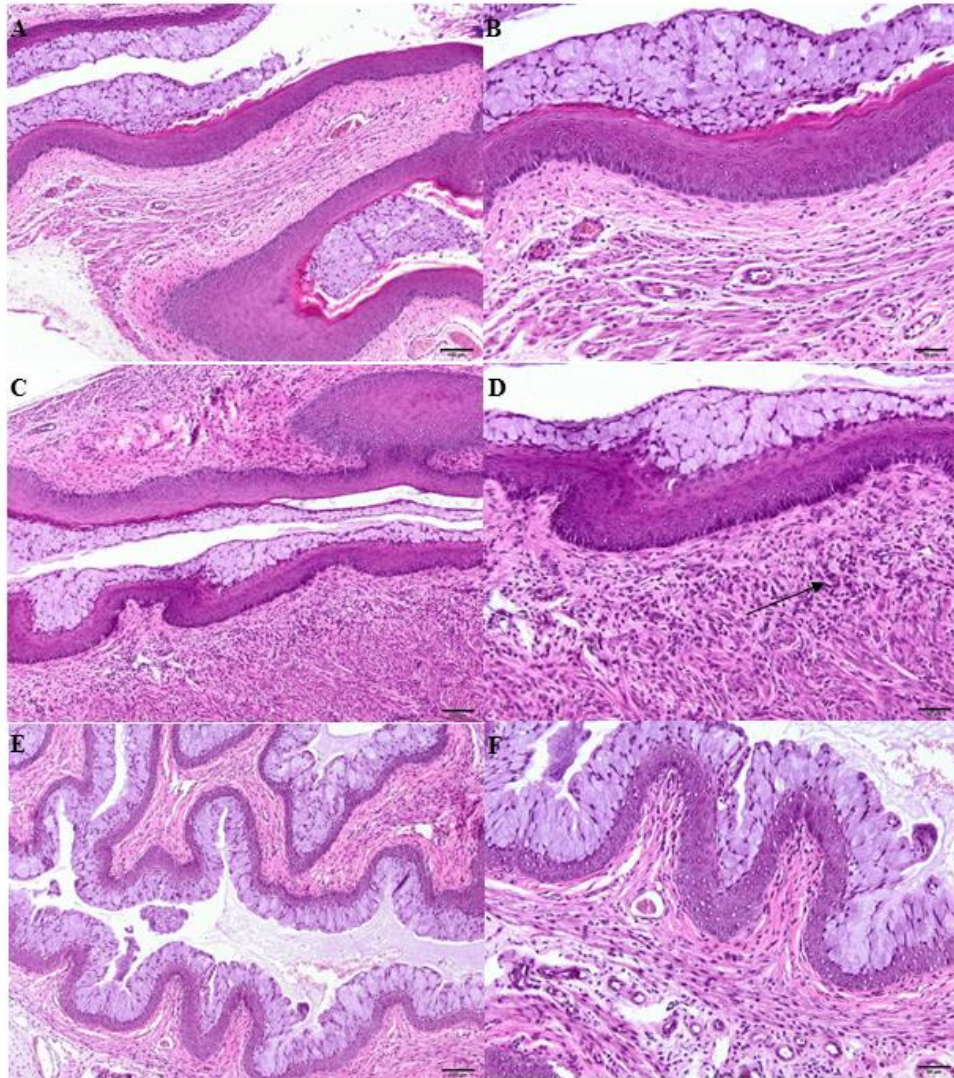

Supplement: S6 Fig — A, B: mice 4 showed minimal epithelial hyperplasia and vascular congestion; C, D: mice 5 presented epithelial hyperplasia and a slight inflammatory infiltrate in the submucosa (arrow). E, F: mice 6 without significant histological lesions. (PDF) [file pone.0195744.s006.pdf]

**S7 Fig**

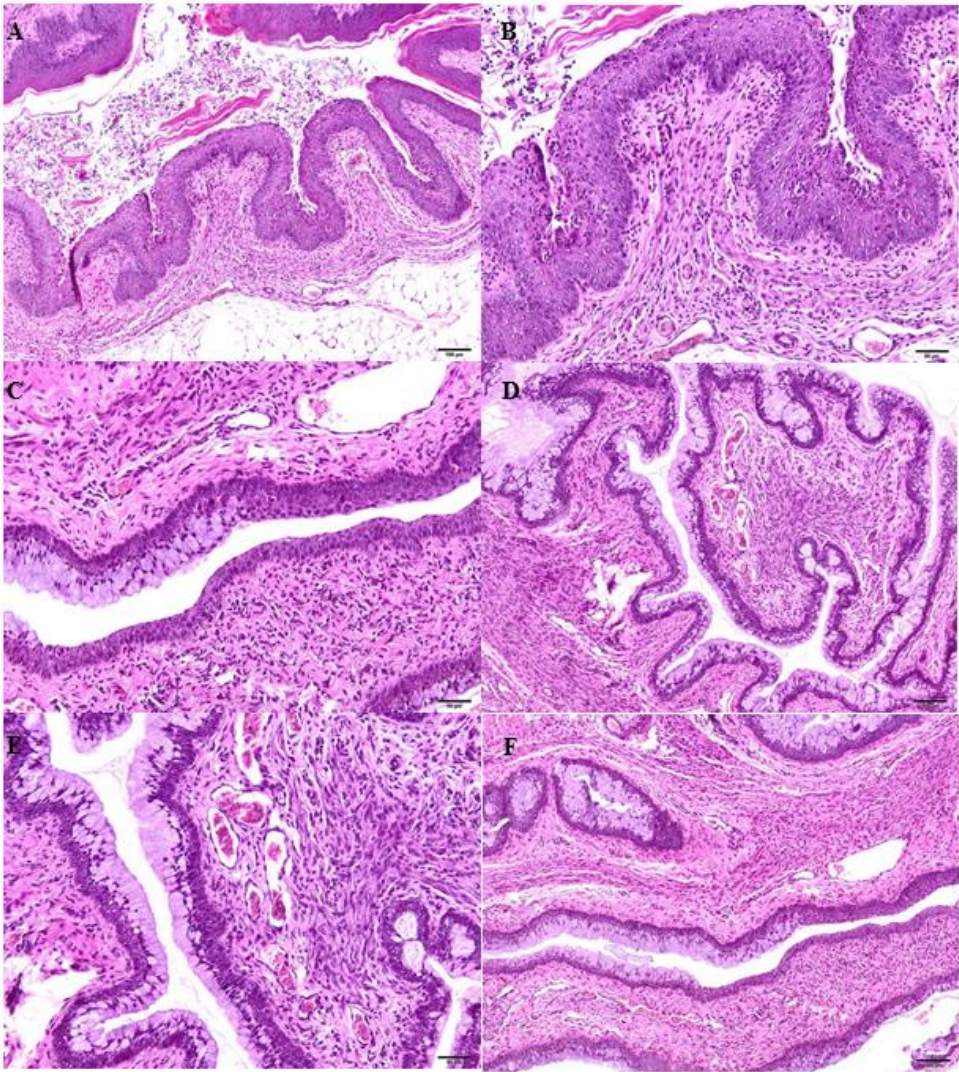

Supplement: S7 Fig — A, B: mice 7 showed minimal inflammatory infiltrate in the epithelium and limited congestion in the submucosa; C, D: mice 8 presented scarce congestion in submucosa; E, F: mice 9 showed minimal inflammatory infiltrate and congestion in the submucosa. (PDF) [file pone.0195744.s007.pdf]

**S8 Fig**

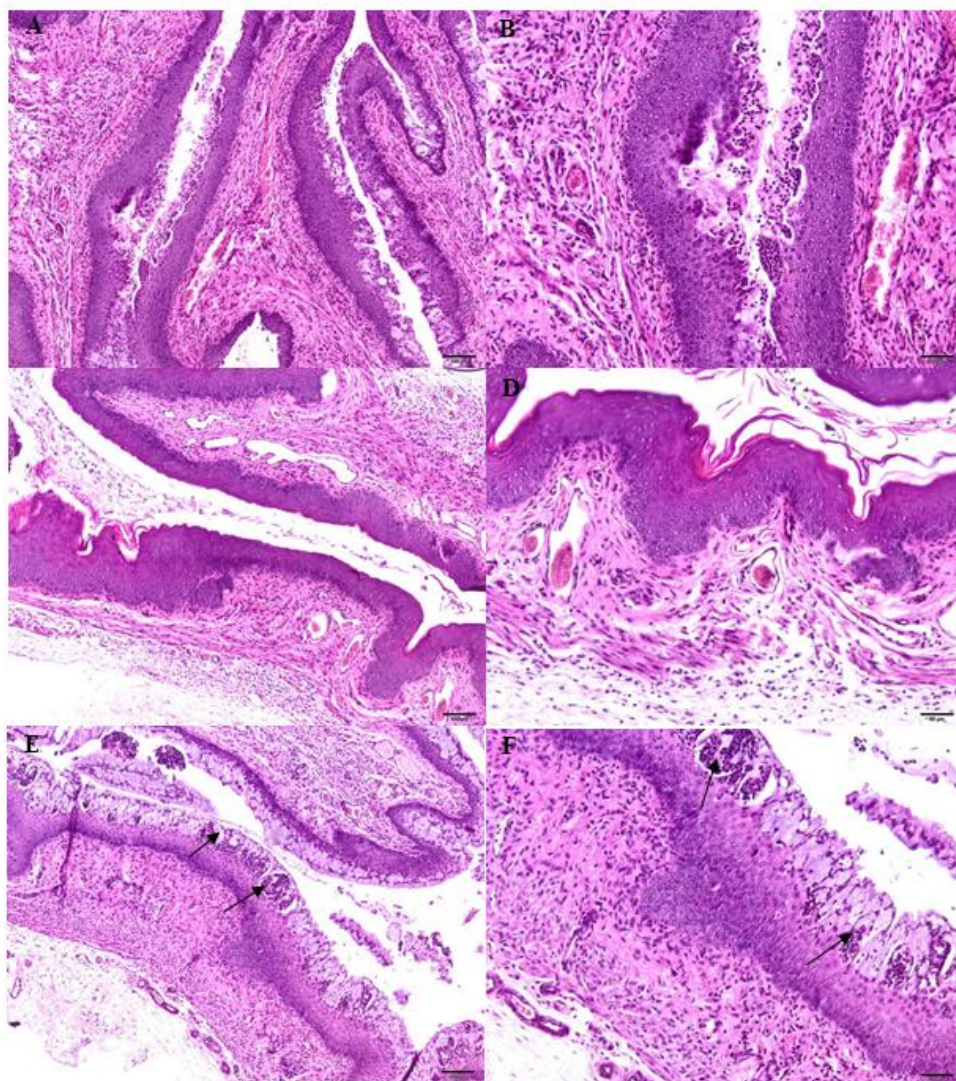

Supplement: S8 Fig — A, B: mice 10 showed severe injury in the epithelium with hyperplasia and inflammatory infiltrate, as well as moderate congestion and inflammatory infiltrate in the submucosa; C, D: mice 11 showed minimal vascular congestion and minimal hyperplasia in the epithelium. E, F: mice 12 presented severe lesions in epithelium with inflammatory infiltrates (arrows) extending throughout submucosa although less evident here. (PDF) [file pone.0195744.s008.pdf]
